# Supplementary material for: Structural basis for calcium-stimulating pore formation of Vibrio α-hemolysin
Source: Nat Commun. 2023 Sep 23;14:5946. doi: 10.1038/s41467-023-41579-x (PMC10517994; doi:10.1038/s41467-023-41579-x)
Supplement: Supplementary file 1 — Supplementary Information [file 41467_2023_41579_MOESM1_ESM.pdf]

Supplementary information

Title: Structural basis for calcium-stimulating pore formation of *Vibrio* α-hemolysin

Authors: Yu-Chuan Chiu[1†], Min-Chi Yeh[2†], Chun-Hsiung Wang[2], Yu-An Chen[1], Hsiang Chang[1], Han-You Lin[3], Meng-Chiao Ho[2,4,5], and Shih-Ming Lin[1,6*]

[1]*Department of Biotechnology and Bioindustry Sciences, National Cheng Kung University, Tainan, Taiwan*

[2]*Institute of Biological Chemistry, Academia Sinica, Taipei, Taiwan*

[3]*Department of Veterinary Medicine, School of Veterinary Medicine, National Taiwan University, Taipei, Taiwan*

[4]*Institute of Biochemical Sciences, National Taiwan University, Taipei, Taiwan*

[5]*Graduate Institute of Biochemistry and Molecular Biology, National Taiwan University Taipei, Taiwan.*

[6]*Institute of Tropical Plant Sciences and Microbiology, National Cheng Kung University, Tainan, Taiwan*

[†]*The authors contributed equally to this work*
**Correspondence: smlin@mail.ncku.edu.tw*

This file includes:
Supplementary Tables 1-5
Supplementary Figures 1-8

Supplementary Table 1. Analysis of domain identity between VcαHL and *V. cholerae* cytolysin (VCC)

**Domain**	**VcαHL** domain range (a.a.)	***V. cholerae* cytolysin** domain range (a.a.)	**Amino acid sequence identity[#]**
All	1-712	1-716	89.66%
Pro domain	1-110	1-110	88.07%
Cytolysin	133-455	137-459	90.71%
Pre-stem loop	277-318	281-322	83.33%
β-trefoil lectin	456-577	460-581	93.44%
β-prism lectin	578-712	582-716	91.11%

[#]The sequence identity is calculated by using protein-protein Basic Local Alignment Search Tool (BLAST)

Supplementary Table 2. X-ray data collection and refinement statistics

	pro-VcαHL (PDB-8JBQ)
**Data collection**	
Space group	$P4_32_12$
Cell dimensions	
a, b, c (Å)	208.83, 208.83, 52.31
α, β, γ (°)	90.00, 90.00, 90.00
Wavelength (Å)	1.00
Resolution (Å)	30.00–2.0 (2.07–2.00)
*R*merge (%)[a]	10.9 (79.0)
I/σ	16.18 (2.31)
CC1/2 (%)	0.998 (0.727)
Completeness (%)	99.9 (99.8)
Redundancy	6.7 (6.1)
**Refinement**	
Resolution (Å)	30.00–2.0
No. reflections	78478 (7731)
*R*work[b] / *R*free (%)[c]	18.02/19.70
No. atoms	
Protein	5,474
SO_4^{2-}	15
Water	620
B-factors (Å^2)	
Protein	37.97
SO_4^{2-}	55.40
Water	41.72
r.m.s deviations	
Bond lengths (Å)	0.002
Bond angles (°)	0.490
Ramachandran plot	
Favored (%)	97.82
Allowed (%)	2.18

*Highest resolution shell is shown in parenthesis.

Supplementary Table 3. Cryo-EM data collection, refinement, and validation statistics

	**VcαHL (EMD-36150) (PDB-8JC7)**
**Data collection**	
EM equipment	Titan Krios
Voltage (kV)	300
Cs (mm)	2.7
Magnification (nominal)	81,000
Detector	K3
Pixel size (Å)	0.5305 (super resolution)
Electron exposure (e^{-}/Å^2)	~ 50
Exposure time (s)	2.15
Frames (no.)	40
Defocus range (μm)	-0.5 ~ -3.0
**Reconstruction**	
Micrographs stacks (no.)	7,96
Final particle images (no.)	142,403
Symmetry imposed	C7
Map final resolution (Å)	2.06
FSC threshold	0.143
Map sharpening B-factor (Å^2)	-65.7
**Refinement**	
Model resolution (Å)	2.1 (unmasked)
FSC threshold	0.143
Map CC (around atoms)	0.91
Model composition	
Non-hydrogen atoms	25,623
Protein residues	3,136
Ligands	14
Waters	591
B factors (Å^2)	
Protein	64.49
Ligands	94.04
Waters	67.91
r.m.s. deviations[a]	
Bond lengths (Å)	0.011
Bond angles (°)	0.596
**Validation**	
MolProbity score	1.42
Clash score	5.75
Ramachandran plot	
Favored (%)	97.47
Allowed (%)	2.53
Outliers (%)	0.00
Rotamer outliers (%)	0.88
C_β deviations	0.00

[a]root-mean-squared deviation (r.m.s. Δ) from target geometries.

Supplementary Table 4. Membrane association affinities for VcαHL and its variants as determined by ELISA analysis

VcαHL	K_d (μM)	Hill slop	B_{max} (AU)	R-squared※
WT	0.02 ± 0.001	1.78 ± 0.104	2.84 ± 0.040	0.992
H415A	0.08 ± 0.003	1.91 ± 0.119	2.73 ± 0.058	0.994
H422A	0.06 ± 0.002	2.25 ± 0.152	2.61 ± 0.047	0.994
H426A	0.04 ± 0.001	2.09 ± 0.145	2.84 ± 0.047	0.992

Values are derived from non-linear regression analysis of 24 data points using binding-saturation functions. The standard errors of the mean (S.E.M.) are provided for error indication.

※ The R-squared values indicate the quality of the fit.

Supplementary Table 5. List of primers utilized in this study

VcαHL		Sequence (5' to 3')
Wild-type	Forward	AAGGAGATATACATATGAATATCAATGAGCCAAGTGGTGAAG#
	Reverse	GGTGGTGGTGCTCGAGGTTCAAATCAAATTGAACCCCTTTC#
H137A	Forward	CTTGCCG**GCG**GTGGCGTTCTACATTAGTGTCAACC
	Reverse	GCCAC**CGC**CGGCAAGGTGCTTGTTTCG
N183A	Forward	TAGGGTTG**CC**CTAGAACGTTCGTTGCAATAC
	Reverse	TCTAGG**GC**AACCCTATAAATCAATGAGATATTG
E185A	Forward	TAACCTAG**C**ACGTTCGTTGCAATACGGAATT
	Reverse	GAACGT**G**CTAGGTTAACCCTATAAATCAATGAG
H415A	Forward	TTATAAA**GC**CTACTATGTGGTCGGTGCTC
	Reverse	TAGTAG**GC**TTTATAAGCACCGTTATAGATTGGG
H422A	Forward	CGGTGCT**GC**TCAGTCCTATCATGGCTTTGAA
	Reverse	GACTGA**GC**AGCACCGACCACATAGTAGTGT
H426A	Forward	GTCCTAT**GC**TGGCTTTGAAAATAGCCCACGTCG
	Reverse	AAGCCA**GC**ATAGGACTGATGAGCACCGACC

The mutated codons are underlined, and the replaced nucleotides are shown in bold.
#The sequence encoding VcαHL gene is indicated by double underline.

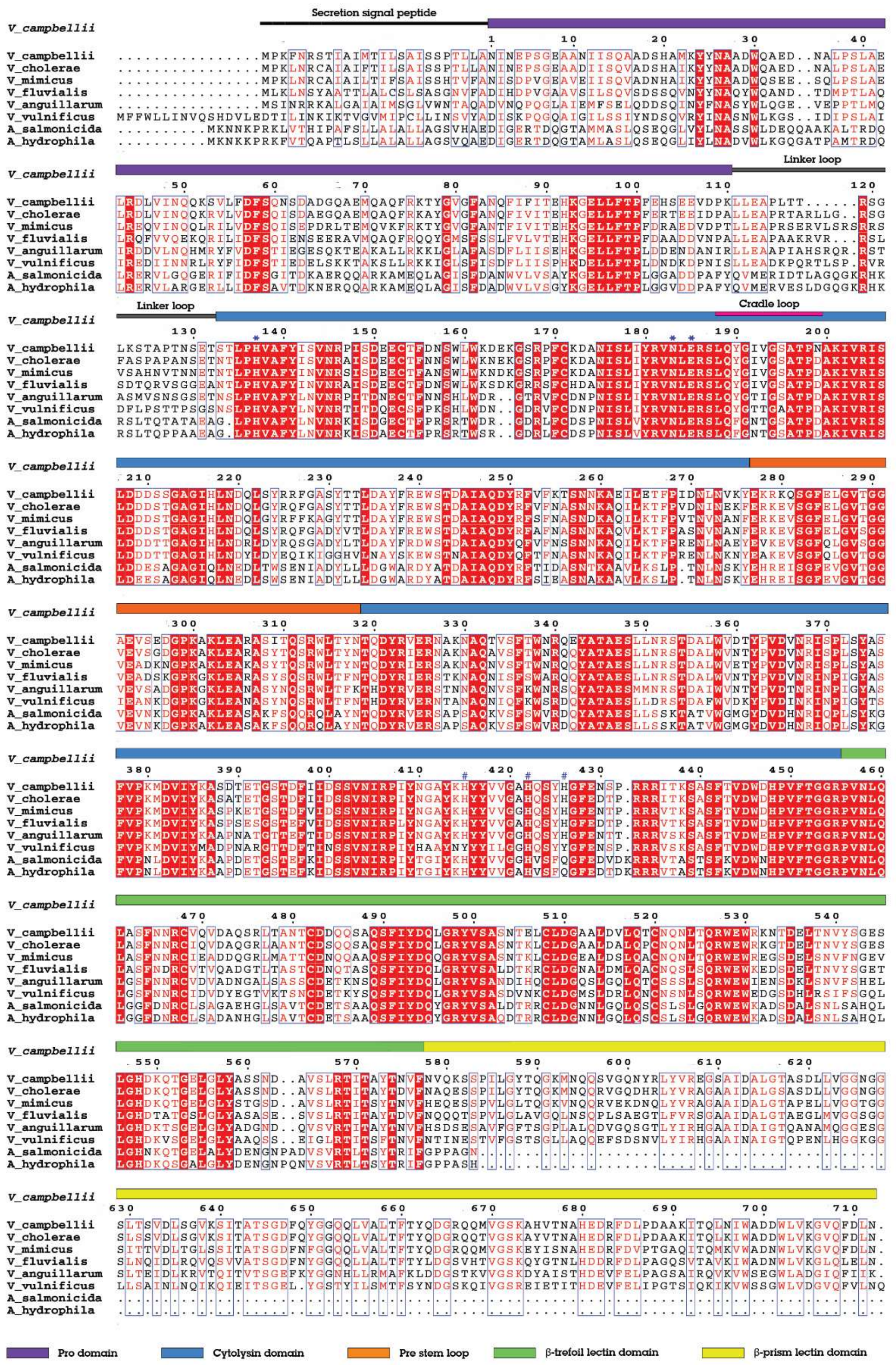

Supplementary Figure 1. Multiple sequence alignment of αHLs from *Vibrio* and *Aeromonas* species. The domain range and residue numbers of VcαHL are labeled above the sequence. Calcium-binding residues are denoted by an asterisk (*), whereas histidine residues located at the rim region, which are embedded in the membrane, are marked with a pound sign (#). The cradle loop region is indicated by a magenta line.

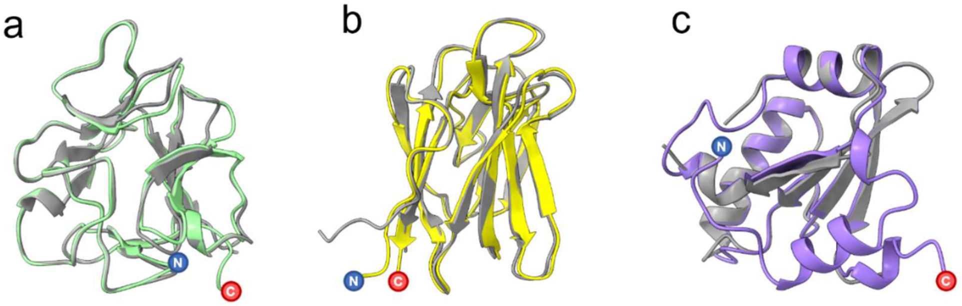

Supplementary Figure 2. Superimposition of individual domains in pro-VcαHL and pro-VCC structures. In each panel, respective domains from pro-VcαHL (color-coded by domain) and pro-VCC (depicted in gray, PDB code: 1XEZ [https://doi.org/10.2210/pdb1XEZ/pdb]) have been superimposed to highlight structural similarities. **(a)** The β-trefoil lectin domains, **(b)** β-prism lectin domains, and **(c)** pro domains. Blue and red circles respectively denote the N- and C-termini of each domain in pro-VcαHL.

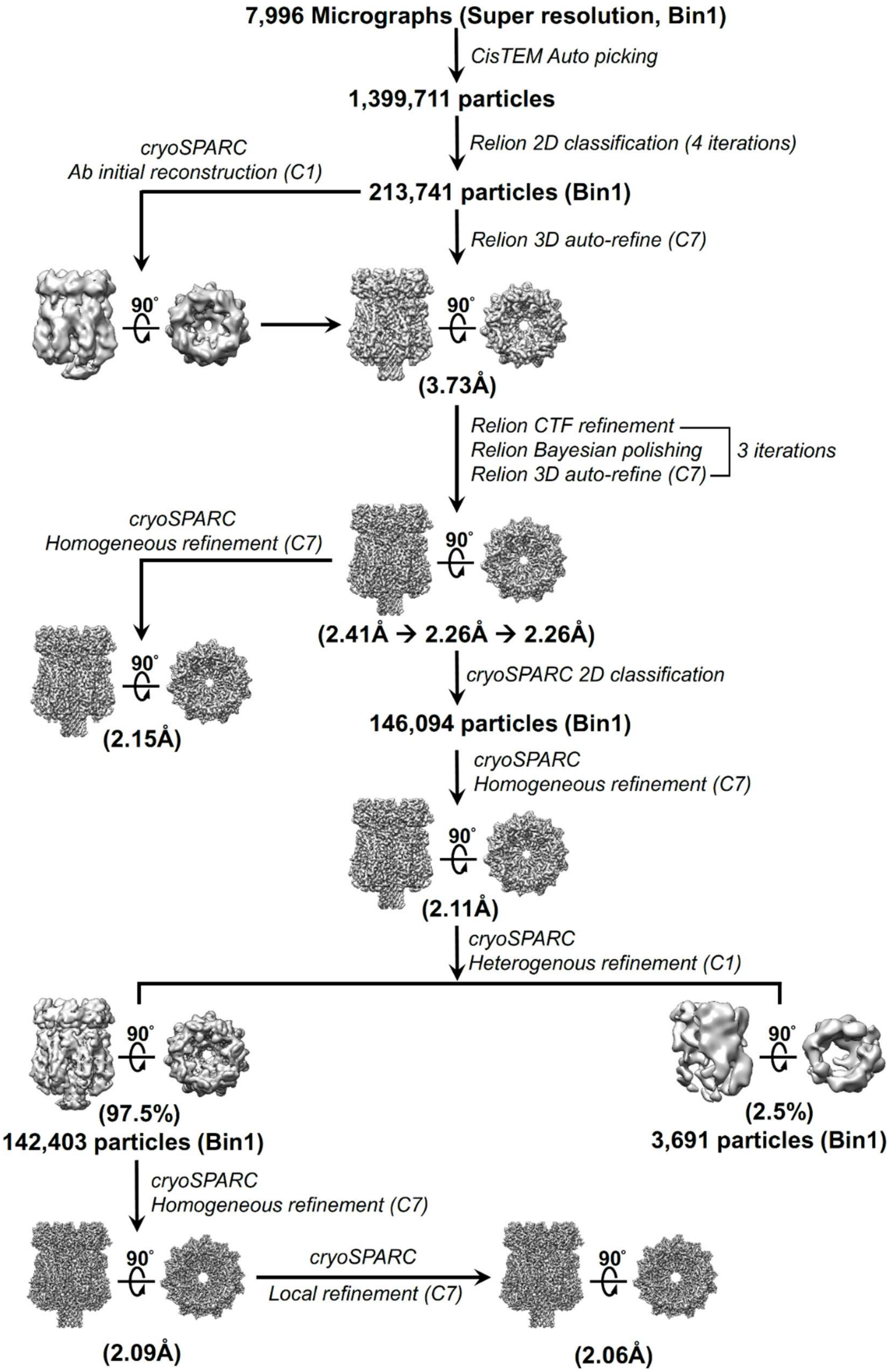

Supplementary Figure 3. Cryo-EM data processing workflow for the assembled VcαHL. This figure demonstrates the sequence of steps that transform raw data into a refined cryo-EM map. Each specific stage is labeled with the corresponding data processing program. The designations C1 and C7 represent the symmetry used during the refinement process.

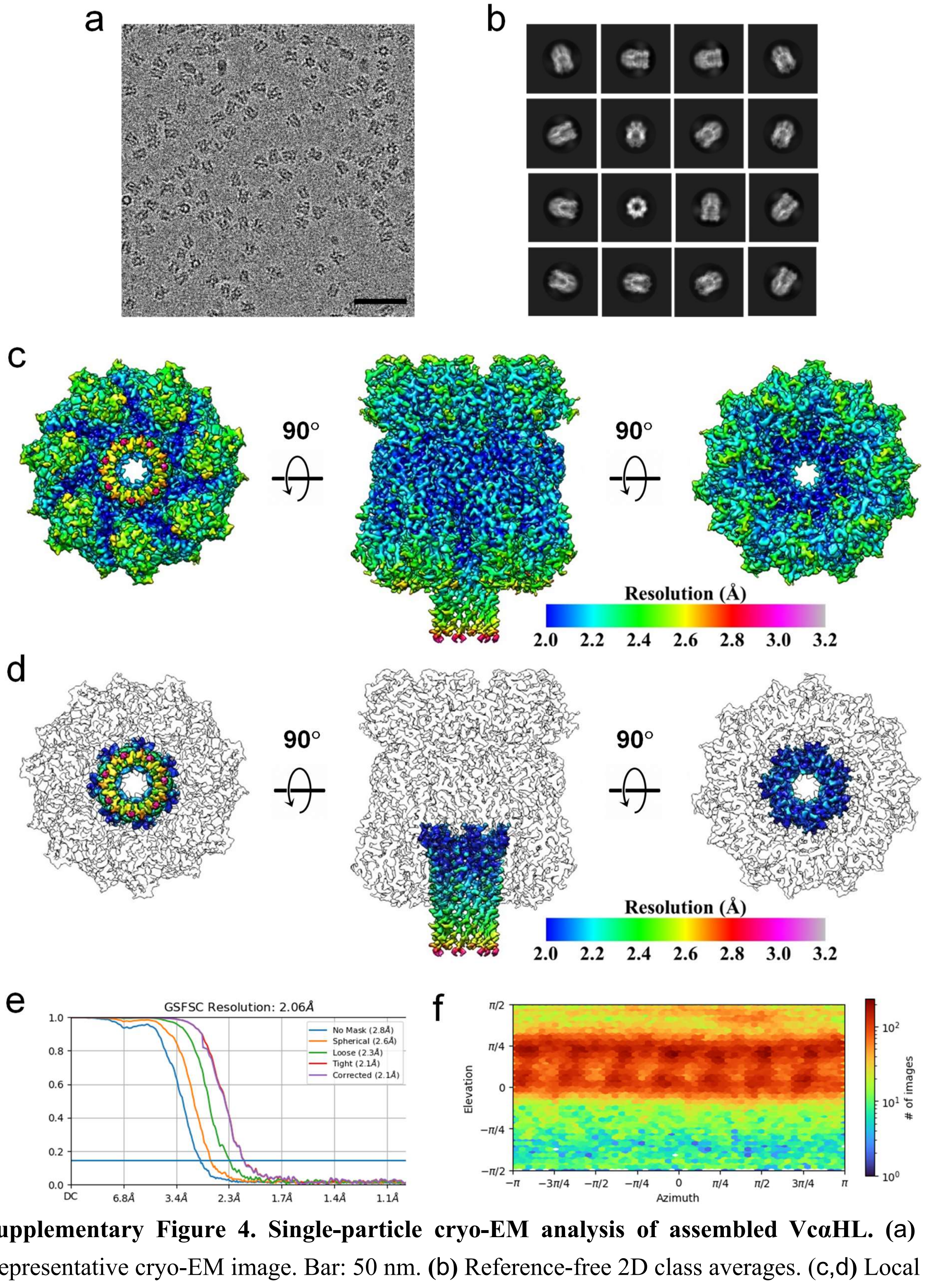

Supplementary Figure 4. Single-particle cryo-EM analysis of assembled VcαHL. (a) Representative cryo-EM image. Bar: 50 nm. **(b)** Reference-free 2D class averages. (c,d**)** Local resolution estimation for the final reconstruction. **(e)** Gold standard FSC plots for the 3D reconstructions. **(f)** Euler angle distribution of particle images.

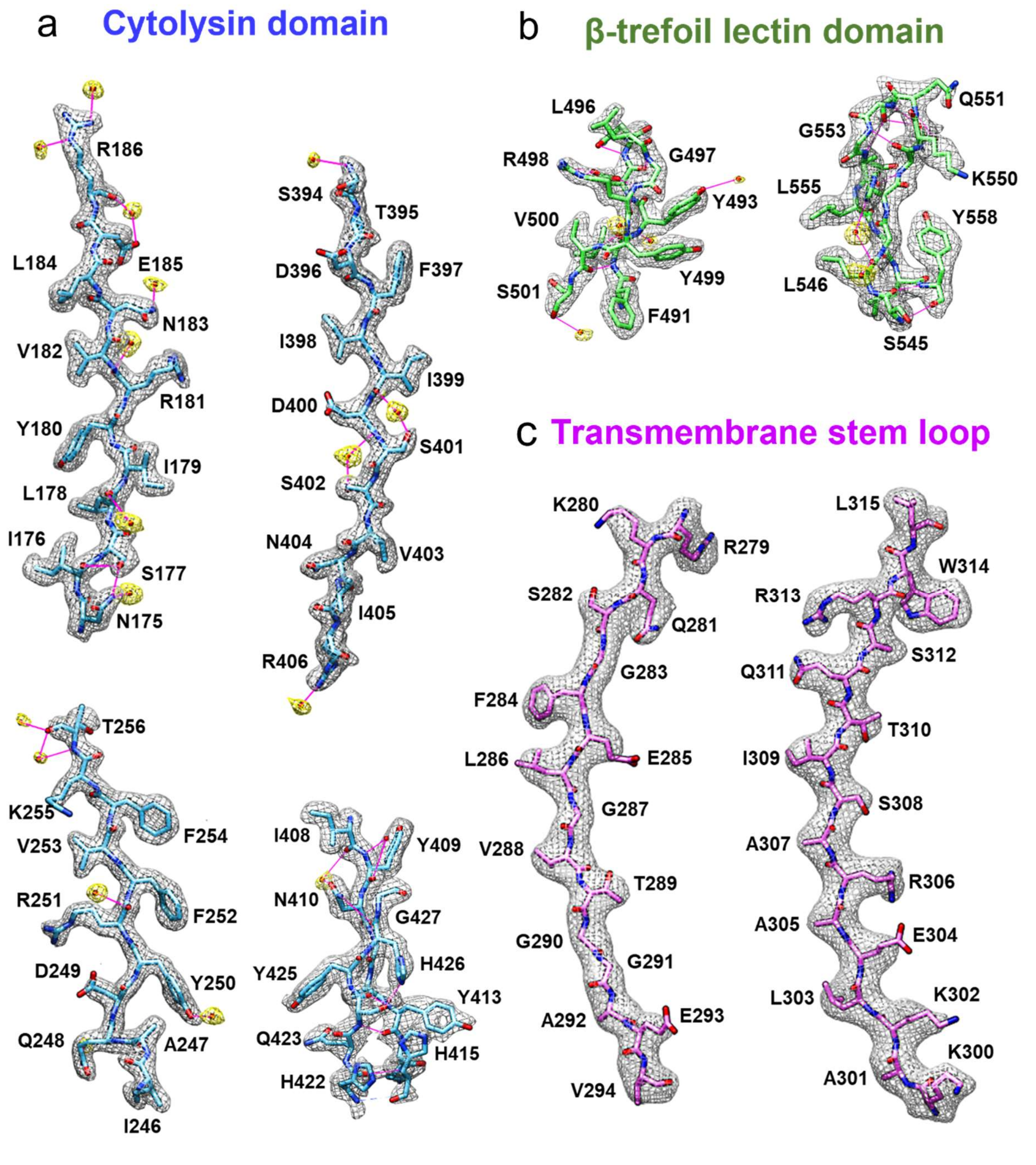

Supplementary Figure 5. Visualization of cryo-EM density and atomic models in the assembled VcαHL complex. Representative regions of cryo-EM density and their corresponding atomic models are shown for **(a)** the cytolysin domain (light blue), **(b)** the beta trefoil lectin domain (light green), and **(c)** the transmembrane stem loop (pink). The cryo-EM densities are represented as a gray mesh, and the residues for each segment are labeled with the amino acid and residue number.

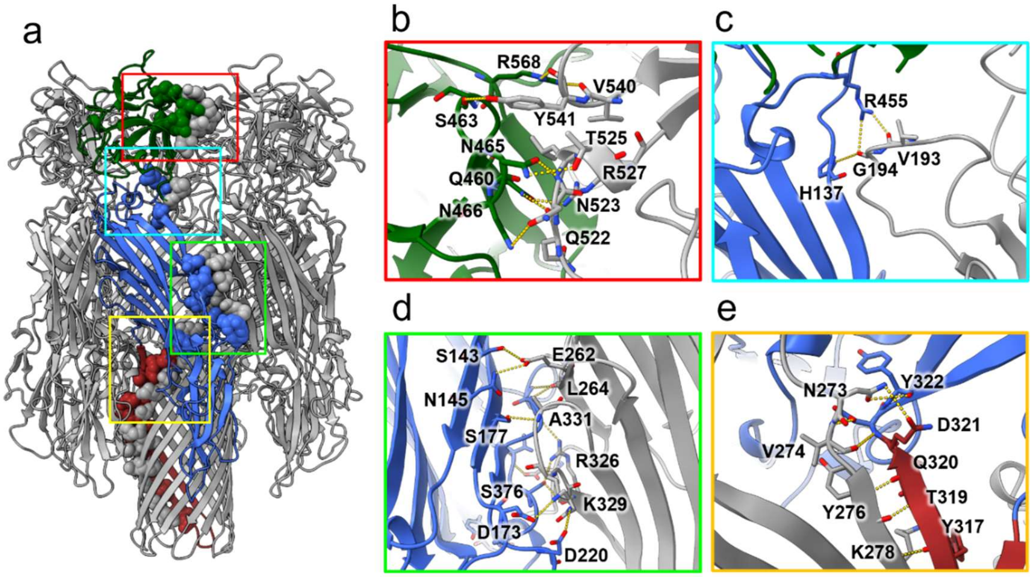

Supplementary Figure 6. Analysis of residue interactions at the subunit interface within the heptameric VcαHL assembly. (a) Residues interacting between chain A and G in the VcαHL assembly, as identified by PDBePISA (Proteins, Interfaces, Structures, and Assemblies), are represented as spheres on the cartoon model of assembled VcαHL. Each distinct domain in chain A is color-coded: green for the β-trefoil lectin domains, blue for the cytolysin domain, and brown for the stem loop region. **(b-e)** Detailed views of the protomer interfaces at the (b**)** β-trefoil lectin domain, (c) cradle loop, (d) cytolysin domain, and (e) entrance region of the transmembrane pore are provided. The colored boxes in a correspond to the regions displayed in (b-e). The residues contributing to inter-subunit hydrogen bonding (indicated by yellow dashed lines) are rendered as sticks and labeled.

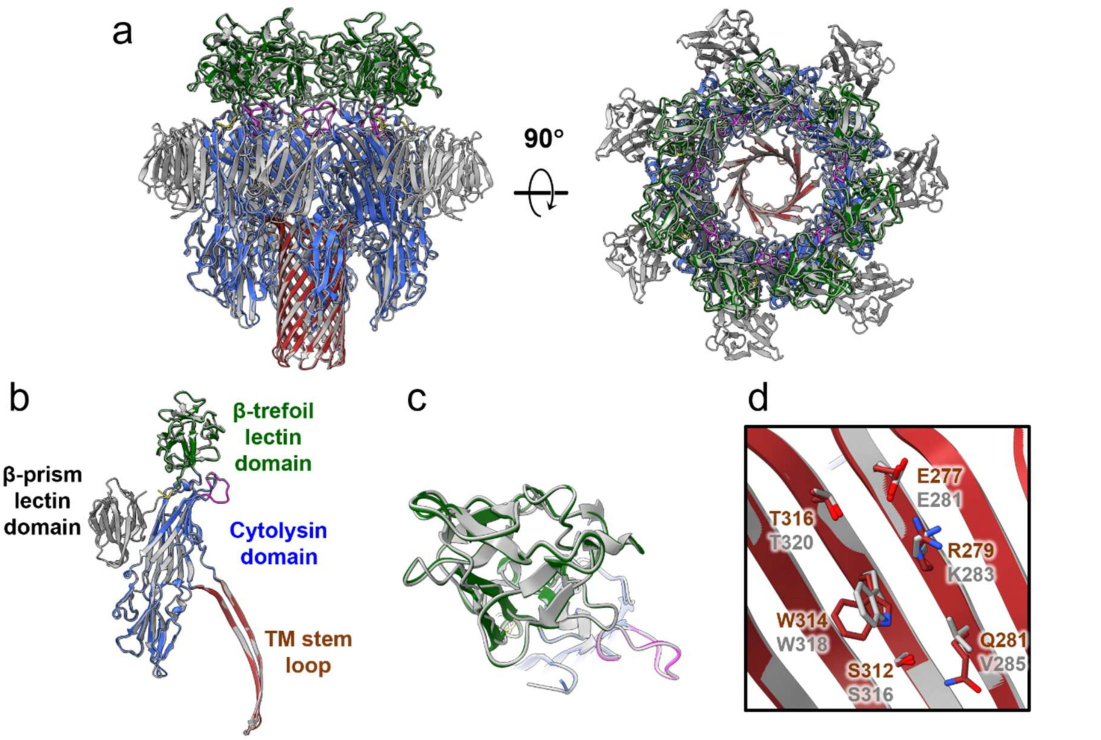

Supplementary Figure 7. Structural comparison of assembled VcαHL and VCC reveals converged structures with distinct tryptophan residues in the pore channel. (a) Superimposed structures of assembled VcαHL (color-coded by domain) and VCC (colored in gray, PDB code: 3O44 [http://doi.org/10.2210/pdb3O44/pdb]). **(b)** Overlapping protomers from VcαHL and VCC display similar conformations, except for discrepancies in the β-prism lectin domains. **(c)** The β-trefoil lectin domain in both assembled VcαHL and VCC aligns to a similar position within the complex. **(d)** Distinctive differences in the pore channel's structure are exposed when comparing assembled VcαHL and VCC, with specific residues shown as sticks and labeled (brown for VcαHL and gray for VCC).

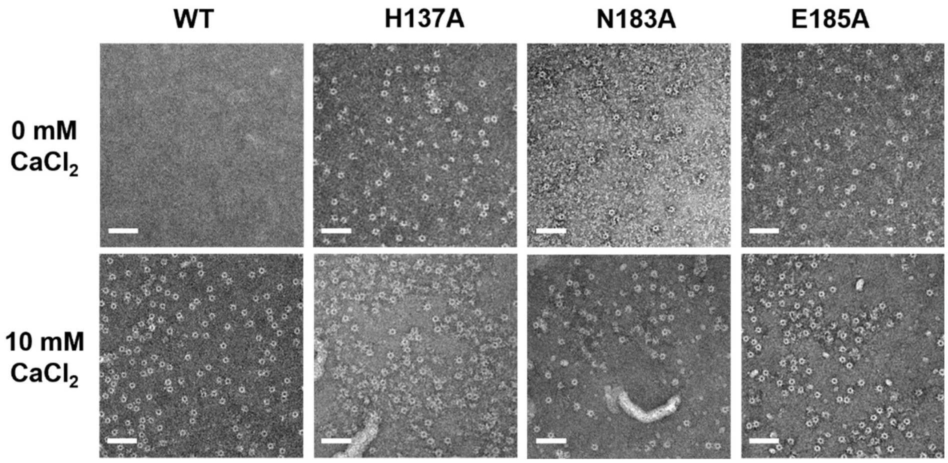

Supplementary Figure 8. Negative-stain TEM images show that mutations at the Ca^{2+}-binding residues impact the calcium-dependency of VcαHL. The wild-type and alanine substitute mutants were treated with liposomal membrane in the presence or absence of Ca^{2+} and visualized by using negative-stain TEM. The scale bar represents 50 nm.
