# Supplementary material for: Structural basis for calcium-stimulating pore formation of Vibrio α-hemolysin
Source: Nat Commun. 2023 Sep 23;14:5946. doi: 10.1038/s41467-023-41579-x (PMC10517994; doi:10.1038/s41467-023-41579-x)
Supplement: Supplementary file 3 — Reporting Summary [file 41467_2023_41579_MOESM3_ESM.pdf]

nature portfolio

Corresponding author(s): Shih-Ming Lin

Last updated by author(s): Sep 1, 2023

Reporting Summary

Nature Portfolio wishes to improve the reproducibility of the work that we publish. This form provides structure for consistency and transparency in reporting. For further information on Nature Portfolio policies, see our Editorial Policies and the Editorial Policy Checklist.

Statistics

For all statistical analyses, confirm that the following items are present in the figure legend, table legend, main text, or Methods section.

n/a	Confirmed	
☐	☒	The exact sample size (n) for each experimental group/condition, given as a discrete number and unit of measurement
☐	☒	A statement on whether measurements were taken from distinct samples or whether the same sample was measured repeatedly
☒	☐	The statistical test(s) used AND whether they are one- or two-sided<br>*Only common tests should be described solely by name; describe more complex techniques in the Methods section.*
☒	☐	A description of all covariates tested
☒	☐	A description of any assumptions or corrections, such as tests of normality and adjustment for multiple comparisons
☐	☒	A full description of the statistical parameters including central tendency (e.g. means) or other basic estimates (e.g. regression coefficient) AND variation (e.g. standard deviation) or associated estimates of uncertainty (e.g. confidence intervals)
☒	☐	For null hypothesis testing, the test statistic (e.g. F, t, r) with confidence intervals, effect sizes, degrees of freedom and P value noted<br>*Give P values as exact values whenever suitable.*
☒	☐	For Bayesian analysis, information on the choice of priors and Markov chain Monte Carlo settings
☒	☐	For hierarchical and complex designs, identification of the appropriate level for tests and full reporting of outcomes
☒	☐	Estimates of effect sizes (e.g. Cohen's d, Pearson's r), indicating how they were calculated

Our web collection on statistics for biologists contains articles on many of the points above.

Software and code

Policy information about availability of computer code

Data collection	X-ray diffraction data were collected using BluIce 5.0, cryo-EM images were acquired using Control Software, EPU-2.7.0
Data analysis	X-ray diffraction data were processed and scaled using HKL2000 v722. Model refinement was performed using the PHENIX suite v1.19, and manual modeling was conducted between refinement cycles using COOT-0.9. Cryo-EM data underwent motion-correction and dose-weighting using MotionCor2-1.4.0. The calculation of the contrast transfer function (CTF) using CTFFIND4.1. Particle picking, extraction, and classification were carried out in cisTEM-1.00 beta and Relion-3.0. The ab initio map generation is conducted by cryoSPARC 4.0.2. All charts are made by using Graphpad Prism 8.0.2. All structural figures and density maps are created by UCSF ChimeraX v1.5. HOLE-2.3.0 is used to analyze the pore channel radius of assembled VcαHL. HOLLOW-1.3 is used to display the channel surface of assembled VcαHL.

For manuscripts utilizing custom algorithms or software that are central to the research but not yet described in published literature, software must be made available to editors and reviewers. We strongly encourage code deposition in a community repository (e.g. GitHub). See the Nature Portfolio guidelines for submitting code & software for further information.

Data

Policy information about availability of data

All manuscripts must include a data availability statement. This statement should provide the following information, where applicable:

- Accession codes, unique identifiers, or web links for publicly available datasets
- A description of any restrictions on data availability
- For clinical datasets or third party data, please ensure that the statement adheres to our policy

The cryo-EM density map has been deposited in the Electron Microscopy Data Bank (EMDB) under accession code, EMD-36150 [https://www.ebi.ac.uk/pdbe/entry/emdb/EMD-36150] (assembled VcαHL). The atomic coordinates have been deposited in the Protein Data Bank (PDB) under accession codes, 8JBQ [https://doi.org/10.2210/pdb8JBQ/pdb] (pro-VcαHL) and 8JC7 [https://doi.org/10.2210/pdb8JC7/pdb] (assembled VcαHL). The source data underlying Figures 1, 2a, 2c, 5c-d, 6b-c and 6e are provided as a Source Data file. The initial model used to determine the crystal structure of pro-VcαHL for molecular replacement was obtained from the Alphafold2 database, AFID: A0A344KRS4 [https://alphafold.ebi.ac.uk/entry/A0A344KRS4]. The model coordinates of VCC used for structural comparison were obtained from PDB, 1XEZ [https://doi.org/10.2210/pdb1XEZ/pdb] (pro-VCC) and 3O44 [http://doi.org/10.2210/pdb3O44/pdb] (assembled VCC).

Research involving human participants, their data, or biological material

Policy information about studies with human participants or human data. See also policy information about sex, gender (identity/presentation), and sexual orientation and race, ethnicity and racism.

Reporting on sex and gender	n/a
Reporting on race, ethnicity, or other socially relevant groupings	n/a
Population characteristics	n/a
Recruitment	n/a
Ethics oversight	n/a

Note that full information on the approval of the study protocol must also be provided in the manuscript.

Field-specific reporting

Please select the one below that is the best fit for your research. If you are not sure, read the appropriate sections before making your selection.

☒ Life sciences ☐ Behavioural & social sciences ☐ Ecological, evolutionary & environmental sciences

For a reference copy of the document with all sections, see nature.com/documents/nr-reporting-summary-flat.pdf

Life sciences study design

All studies must disclose on these points even when the disclosure is negative.

Sample size	For all our biochemical experiments, we chose a sample size with three independent replicates. This decision was driven by statistical rigor. The use of three independent replicates allowed us to average out the data points, minimizing potential biases and providing a more accurate representation of the true value. Averaging the results from these replicates helped in reducing noise. By having each sample prepared in triplicate, we ensured that any random anomalies or minor inconsistencies during the experiment did not skew the results.
Data exclusions	No data were excluded from the analyses
Replication	All experiments were performed independently at least three times to ensure consistency and reproducibility of the results.
Randomization	At the crystal structure refinement process, a small percentage (typically 5-10%) of the reflection data is randomly selected and set aside to calculate Rfree. These reflections are excluded from subsequent refinement steps to maintain their independence from the model. By comparing the Rfree value with Rwork (calculated from the remaining 90-95% of reflections), we can gauge the quality of the model and the likelihood of overfitting. For cryo-EM single particle reconstitution, particles are randomly divided into two group to calculate the half-maps. By randomizing and independently processing two subsets of data, cryo-EM analysts can ensure that the resolution estimate from the FSC is not biased by any potential artifacts or over-processing.
Blinding	Blinding was not applicable to this study because the primary outcome of the measurements was objective and relied on instrumental data collection methods. Given this design, the potential for observer bias or subjective interpretation is minimal.

Reporting for specific materials, systems and methods

We require information from authors about some types of materials, experimental systems and methods used in many studies. Here, indicate whether each material, system or method listed is relevant to your study. If you are not sure if a list item applies to your research, read the appropriate section before selecting a response.

Materials & experimental systems

n/a	Involved in the study	
☐	☒	Antibodies
☒	☐	Eukaryotic cell lines
☒	☐	Palaeontology and archaeology
☒	☐	Animals and other organisms
☒	☐	Clinical data
☒	☐	Dual use research of concern
☒	☐	Plants

Methods

n/a	Involved in the study	
☒	☐	ChIP-seq
☒	☐	Flow cytometry
☒	☐	MRI-based neuroimaging

Antibodies

Antibodies used

Antibodies used for this project to detect recombinant VcαHL are a mouse anti-his primary antibody (Anti-Histidine Tagged Antibody, clone HIS.H8, Cat. #05-949, Lot. 3660392, 1:8000 dilution, Millipore) and a goat anti-mouse IgG secondary antibody conjugated with alkaline phosphatase (PN NEF824001EA, Lot. 120015, 1:10000 dilution, PerkinElmer)

Validation

According to the Certificate of Analysis found on manufacturer's website, the anti-his primary antibody is validated by Western Blot Analysis: 0.5-2 μg/mL of this lot detected His tagged Akt1/PKBα in RIPA lysates of transfected HeLa cells. The specificity of this primary antibody is recognizing tag sequence HHHHHH on either the N- or C-terminus of any recombinant fusion protein; no other amino acids surrounding the 6x-His tag sequence are required for the recognition of the 6x-His tag. The secondary antibody is affinity purified antibody, isolated from a pool od serum goats immunized with purified Mouse IgG was labeled with phosphatase using a modified glutaraldehyde procedure. The specificity is tested by immunoelectrophoresis, gel diffusion & ELISA techniques as applicable. This product reacts specifically with Mouse IgG and may recognize other immunoglobulin types that have light chains in common with IgG. Cross-reactivity to human serum has been minimized using affinity procedures. No antibody is detectable to non-immunoglobulin serum components.
